# Aη-α and Aη-β peptides impair LTP ex vivo within the low nanomolar range and impact neuronal activity in vivo

**DOI:** 10.1186/s13195-021-00860-1

**Published:** 2021-07-08

**Authors:** Maria Mensch, Jade Dunot, Sandy M. Yishan, Samuel S. Harris, Aline Blistein, Alban Avdiu, Paula A. Pousinha, Camilla Giudici, Marc Aurel Busche, Peter Jedlicka, Michael Willem, Hélène Marie

**Affiliations:** 1 Université Côte d’Azur, CNRS, IPMC, 660 Route des Lucioles, 06560 Valbonne, France; 2 Munich Cluster for Systems Neurology (SyNergy), Munich, Germany; 3 UK Dementia Research Institute at UCL, University College London, London, WC1E 6BT UK; 4 Institute of Clinical Neuroanatomy, Goethe University, Frankfurt am Main, Germany; 5 German Center for Neurodegenerative Diseases (DZNE-Munich), 81377 Munich, Germany; 6 Institute of Neuroscience, Technische Universität München, 80802 Munich, Germany; 7 Faculty of Medicine, ICAR3R - Interdisciplinary Centre for 3Rs in Animal Research, Justus-Liebig-University, Giessen, Germany; 8 Biomedical Center (BMC), Ludwig-Maximilians-University Munich, 81377 Munich, Germany

**Keywords:** APP processing, Synaptic plasticity, Electrophysiology, Hippocampus, Alzheimer

## Abstract

**Background:**

Amyloid precursor protein (APP) processing is central to Alzheimer’s disease (AD) etiology. As early cognitive alterations in AD are strongly correlated to abnormal information processing due to increasing synaptic impairment, it is crucial to characterize how peptides generated through APP cleavage modulate synapse function. We previously described a novel APP processing pathway producing η-secretase-derived peptides (Aη) and revealed that Aη–α, the longest form of Aη produced by η-secretase and α-secretase cleavage, impaired hippocampal long-term potentiation (LTP) ex vivo and neuronal activity in vivo.

**Methods:**

With the intention of going beyond this initial observation, we performed a comprehensive analysis to further characterize the effects of both Aη-α and the shorter Aη-β peptide on hippocampus function using ex vivo field electrophysiology, in vivo multiphoton calcium imaging, and in vivo electrophysiology.

**Results:**

We demonstrate that both synthetic peptides acutely impair LTP at low nanomolar concentrations ex vivo and reveal the N-terminus to be a primary site of activity. We further show that Aη-β, like Aη–α, inhibits neuronal activity in vivo and provide confirmation of LTP impairment by Aη–α in vivo.

**Conclusions:**

These results provide novel insights into the functional role of the recently discovered η-secretase-derived products and suggest that Aη peptides represent important, pathophysiologically relevant, modulators of hippocampal network activity, with profound implications for APP-targeting therapeutic strategies in AD.

**Supplementary Information:**

The online version contains supplementary material available at 10.1186/s13195-021-00860-1.

## Background

The amyloid precursor protein (APP) is a transmembrane protein that is highly expressed in neurons of the developing and adult brain. Due to its location at synaptic and perisynaptic sites [1], it is ideally positioned to regulate synaptic signaling. At these sites, APP is cleaved into a variety of secreted and intracellular peptides through the action of different proteases [2]. We previously reported the discovery of an additional APP processing pathway involving a novel cleavage site, N-terminal to the β-secretase-1 (BACE1) cleavage site. This site is targeted by an enzymatic process that we named η-secretase and potentially mediated by membrane types 1 and 5 matrix metalloproteinases (MT1-MMP and MT5-MMP) [3]. To corroborate this finding, and provide a first information on the importance of this new APP processing pathway in AD pathogenesis, another study showed that removal of MT5-MMP in the 5XFAD mouse model of AD alleviated several hallmarks of AD pathology, including preservation of hippocampal LTP [4]. APP processing via this pathway leads to the generation of two secreted Aη peptides: a longer form Aη–α and a comparatively shorter form Aη–β that are generated by η-secretase cleavage and subsequent α-secretase or β-secretase cleavage, respectively [3]. APP cleavage is a physiological process which occurs throughout life, with Aη peptides being detected both in adult healthy human and rodent brain tissue [3]. Aη peptides are present in the cerebrospinal fluid (CSF) of healthy humans and, notably, exceed Aβ levels fivefold. Their preponderance suggests that Aη peptides might act as endogenous modulators of neuronal network activity, although this hypothesis remains to be proven. Furthermore, since pathological alterations in APP processing are crucially involved in the etiology of AD [2], unravelling the function of Aη peptides might help to elucidate the pathophysiological mechanisms of homeostatic failure, including defective synapse communication, underlying cognitive decline typical of AD [5–7]. Finally, BACE1 inhibition is currently being evaluated as a potential therapeutic strategy to lower Aβ load in AD patients and prevent cognitive decline. However, we previously demonstrated that BACE-1 inhibition increases levels of Aη peptides [3]. It will thus be important to thoroughly identify their neuromodulatory potential in order to avoid adverse side effects due to this type of treatment.

Beyond the discovery of these Aη peptides, our initial work provided the first insights on their impact on neuron function. Then, we observed that both cell-produced recombinant Aη–α (recAη–α) and synthetic Aη–α (sAη–α) lowered hippocampal long-term potentiation (LTP) ex vivo and reduced spontaneous somatic calcium transients of hippocampal neurons in vivo [3], while recombinant cell-produced Aη–β (recAη–β) did not have any impact on these parameters. Here, we combined ex vivo field electrophysiology, in vivo multiphoton calcium imaging, and in vivo electrophysiology to further characterize the impact of both Aη–α and Aη–β on hippocampal function.

## Methods

### Animals

For ex vivo electrophysiology recordings, 4–8 weeks old male RjOrl:SWISS mice were used, with the exception of one experiment that was performed in 4–8 weeks old male C57Bl/6 mice, as noted in the results section. Experiments were conducted according to the policies on the care and use of laboratory animals stipulated by the ministries of research of the different countries in compliance with the European Communities Council Directive (2010/63). All efforts were made to minimize animal suffering and reduce the number of animals used. The animals were housed three to six per cage under controlled laboratory conditions with a 12-h dark-light cycle and temperature of 22 ± 2 °C. Animals had free access to standard rodent diet and tap water.

For in vivo LTP experiments, all injections and recordings were performed on adult male Sprague-Dawley rats (450–650 g). The experiments were performed in accordance with local institutional and governmental regulations regarding the use of laboratory animals at the University of Frankfurt as approved by the Regierungspräsidium Darmstadt and the animal welfare officer responsible for the institution.

For in vivo calcium imaging experiments, male and female C57Bl/6 mice (~P40) were used. Experiments were conducted in compliance with institutional (Technische Universität München) animal welfare guidelines and approved by the state government of Bavaria, Germany.

### Peptides

Synthetic Aη peptides were obtained from Peptide Specialty Laboratories (PSL GmbH; Heidelberg, Germany) and consisted of the following sequences:

- Synthetic Aη–α (sAη–α, 108 amino acids) sequence:
  MISEPRISYGNDALMPSLTETKTTVELLPVNGEFSLDDLQPWHSFGADSVPANTENEVEPVDARPAADRGLTTRPGSGLTNIKTEEISEVKMDAEFRHDSGYEVHHQK
- Synthetic Aƞ–β (sAη–β, 92 amino acids) sequence:
  MISEPRISYGNDALMPSLTETKTTVELLPVNGEFSLDDLQPWHSFGADSVPANTENEVEPVDARPAADRGLTTRPGSGLTNIKTEEISEVKM
- Synthetic N-term sAη (sAη–NT, 46 amino acids) sequence:
  MISEPRISYGNDALMPSLTETKTTVELLPVNGEFSLDDLQPWHSFG
- Synthetic C-term sAη–β (sAη–β–CT; 46 amino acids) sequence:
  ADSVPANTENEVEPVDARPAADRGLTTRPGSGLTNIKTEEISEVKM

The peptides were dissolved in dimethyl sulfoxide (DMSO) at 100 μM and placed at − 80 °C for long-term storage. For ex vivo electrophysiology, on day of experiment, aliquots were further diluted in artificial cerebrospinal fluid (aCSF) (see below) to the required concentration (1–100 nM).

For in vivo electrophysiology, aliquots were further diluted on day of experiment in phosphate buffered saline (PBS) to the required concentration (1 μM).

For in vivo calcium imaging, the peptides were combined with Ringer’s solution (see below) on day of experiment to 100 nM.

Recombinant Aη peptides were generated and purified as described previously [3]. Briefly, for the expression of Aη-α and Aη-β in CHO cells, the complementary cDNAs of the respective fragments were amplified by PCR and subcloned into the pSecTag2A vector (Invitrogen) that features an N-terminal secretion signal. CHO cells were cultured in DMEM with 10% FCS and non-essential amino acids. Transfections were carried out using Lipofectamine2000 (Invitrogen) according to the manufacturer’s instructions. The next day media was changed to OPTIMEM (Invitrogen) and the serum-free conditioned media of the transfected cells, expressing the recombinant Aη peptides, were collected after 20 h. Up to 1 l of C-terminally HIS-tagged peptides was collected and filtered (0.2 μM; Tabletop filter from Millipore). The filtrate was purified by anion exchange chromatography using *HiTrap* columns for small-scale protein purification on Äkta system (Cytiva; Ni-NTA). Positive fractions were pooled and the elution buffer was exchanged and concentrated using an Amicon Ultra Centrifugal filter (PLBC Ultracel-PL membrane, 3 kDa MWCO) with 3 volumes of aCSF. The protein concentration was measured based on the OD280 with a Nanodrop device (Thermo Fisher) and calculated for each protein based on the molecular weight of the nonglycosylated peptide including the myc-HIS tag. The preparation was diluted to a final concentration of 10 nM in ACSF on the day of the experiment.

### Biochemical analysis of Aη peptides

Protein concentration of the purified peptides was measured with a Nanodrop spectrophotometer (Thermo Fisher Scientific, Germany) and the molar concentration was calculated and adjusted according to the molecular weight of the peptides (recAη-β MW: 13120.43; recAη-α MW: 15057.45). Peptides were stored until use in a − 80 °C freezer. For quality control, 1 μg of recombinant proteins and synthetic peptides were separated on a Tris-Tricine gel (10–20%, Thermo Fisher Scientific, Germany), stained with GelCode Blue stain, and imaged with an ImageQuant 800 system (Amersham, Germany).

### Ex vivo electrophysiology

Mice were culled by cervical dislocation and hippocampi were dissected and incubated for 5 min in ice-cold oxygenated (95% O_2_/5% CO_2_) cutting solution (in mM): 206 sucrose, 2.8 KCl, 1.25 NaH_2_PO_4_, 2 MgSO_4_, 1 MgCl_2_, 1 CaCl_2_, 26 NaHCO_3_, 10 glucose, 0.4 sodium ascorbate, oxygenated with 95% O_2_ and 5% CO_2_ (pH 7.4). Hippocampal slices (350 μm) were cut on a vibratome (Microm HM600V, Thermo Scientific, France). For recovery, slices were then incubated in oxygenated aCSF for 1 h at 37 ± 1 °C and then stored at room temperature until used for recordings. aCSF composition was (in mM) 124 NaCl, 2.8 KCl, 1.25 NaH_2_PO_4_, 2 MgSO_4_, 3.6 CaCl_2_, 26 NaHCO_3,_ 0.4 sodium ascorbate, 10 glucose, oxygenated with 95% O_2_ and 5% CO_2_, and pH 7.4. All chemicals were from Sigma-Aldrich (Saint-Quentin Fallavier, France). Recordings for all experiments were done at 27 ± 1 °C in a recording chamber on an upright microscope with IR-DIC illumination (SliceScope, Scientifica Ltd., UK). Field recordings were performed using a Multiclamp 700B amplifier (Molecular Devices, San Jose, CA, USA), under the control of pClamp10 software (Molecular Devices, San Jose, CA, USA). Data analysis was executed using Clampfit 10 software (Molecular Devices, San Jose, CA, USA). Field excitatory post-synaptic potentials (fEPSPs) were recorded in the stratum radiatum of the CA1 region (using a glass electrode filled with 1 M NaCl and 10 mM 4-(2-hydroxyethyl)-1-piperazineethanesulfonic acid (HEPES), pH 7.4). The stimuli were delivered to the Schaffer collateral pathway by placing a monopolar glass electrode (capillary Glass, 1.5 mm outer diameter, 0.84 mm inner diameter, WPI, France, filled with aCSF) in the stratum radiatum. fEPSP response was set to approximately 30% of the maximal fEPSP response i.e. approx. 0.2–0.3 mV, with stimulation intensity 10 μA ± 5 μA delivered via stimulation box (ISO-Flex, A.M.P.I. Inc., Israel). Electrodes were placed superficially to maximize exposure to peptides. Slices were perfused with oxygenated aCSF. The baseline fEPSP was obtained by stimulating at 0.066 Hz (1 stimulation/ 15 s). A stable baseline of a fEPSP was first obtained in control conditions (at least 10 min). Then, synthetic or recombinant peptides were applied for at least 15 minutes (for 100 nM data) or 20 min (for other peptide concentrations) to ensure consolidation of baseline prior to LTP induction. If the baseline was not consolidated within 45 min after peptide application, the slice was discarded. Upon confirmation of this stable baseline, LTP was then induced. The peptide was also recirculated throughout the 1-h recording after induction. LTP was induced by a high-frequency stimulation (HFS) protocol: 2 pulses at 100 Hz for 1 s with a 20-s inter-stimulus interval (ISI). “Control” LTP experiments (aCSF only, no application of peptide) were routinely performed interleaved with peptide application during the same experimental period (i.e., same experimental batch on the same batch of mice, on the same electrophysiology rigs, by the same experimenter within the same continuous timeframe).

For all LTP recordings, only the first third of the fEPSP slope was analyzed to avoid population spike contamination. For LTP time-course and bar graph analyses, the first third of the fEPSP slope was calculated in the baseline condition and at 45–60 min post-induction in each recording. The average baseline value was normalized to 100% and values at 45–60 min post LTP induction were normalized to this baseline average (1-min bins).

For paired-pulse ratios (PPRs), two stimuli were delivered at 100, 200, and 300 ms inter-stimulus intervals (ISI). PPRs were calculated as the average of fEPSP2 slope/fEPSP1 slope (10 sweeps average per ISI). Recordings of control (aCSF only) and peptide conditions were interleaved within the same day.

The input/output (I/O) curves were generated by calculating the fEPSP slope corresponding to a given fiber volley (FV) amplitude ranging from 0.1 to 0.4 mA in increments of 0.1 mA measuring 10 sweeps averages. This protocol was first done under aCSF and slices then perfused for 20 min in aCSF either with or without the peptide before repeating the protocol, as within slice control. Input/output graphs compared the fEPSP slope corresponding to the fiber volley measurements at both time points.

### In vivo electrophysiology

Urethane (Sigma-Aldrich GmbH, Munich, Germany) solution was used to anesthetize the animals with an initial injection (2 g/kg body weight) applied intraperitoneally. Supplemental doses (0.2–0.5 g/kg) were injected subcutaneously until the interdigital reflex could no longer be triggered. The body temperature of the animal was constantly controlled through a rectal probe and maintained at 36.5–37.5 °C using a heating pad. For local anesthesia of the scalp, prilocaine hydrochloride with adrenalin 1:200,000 (Xylonest 1%, AstraZeneca GmbH, Wedel, Germany) was injected subcutaneously at the site of incision. The head of the anesthetized rat was placed into a stereotaxic frame for accurate insertion of electrodes and injection cannula. Using standard surgical procedures, we drilled the stimulation and recording holes and removed the dura mater. A tungsten recording micro-electrode glued to a 10-μl Hamilton series syringe was lowered unilaterally into the dentate gyrus hilus (2.5 mm lateral and 3.8 mm posterior to bregma), and a bipolar concentric stimulating electrode (World Precision Instruments, Germany) was lowered unilaterally into the perforant path (4.5 mm lateral to lambda), while monitoring the laminar profile of the response. Current pulses (30–800 μA, 0.1–0.2 ms duration) were generated by a stimulus generator (STG1004, Multichannel Systems, Reutlingen, Germany). The recorded fEPSPs were first amplified (P55 preamplifier, Grass Technologies, West Warwick, RI, USA) and then digitized at 10 kHz for visualization and offline analysis (Digidata 1440A, Molecular Devices, San Jose, CA, USA). The analysis of electrophysiological data was executed using Clampfit 10.2 software (Molecular Devices, San Jose, CA, USA) as well as custom MATLAB scripts (The MathWorks, Natick, MA, USA). As a measure of synaptic LTP, we compared responses with baseline stimulation (at 0.1 Hz) prior to theta-burst stimulation (TBS) with responses subsequent to TBS. At the start of each experiment, stable baselines were recorded. Then, the experimental solution was injected into the hippocampus. In each experiment, injections of 1 μl of sAη–α or sAη–β–CT (1 μM) were delivered from the Hamilton syringe attached to a microinjection unit (Model 5000, Kopf Instruments, Tujunga, CA, USA). Intradentate injections of fluid led to a typical temporary reduction in the fEPSP slope and amplitude, probably caused by changes in extracellular resistivity [8]. The degree of response suppression and recovery can be seen in LTP graphs. After a baseline period of 20 min, LTP was induced using a standard TBS protocol: six series of six trains of six pulses at 400 Hz, with 0.2 s between trains and 20 s between series. Both the pulse width and the stimulus intensity during TBS were doubled in comparison to baseline recordings. The LTP was followed for 60 min using the baseline stimulation protocol. For the analysis of the slope of the fEPSP, only the early component of the waveform, which is not affected by the population spike, was used. LTP in Fig. 5b, c was calculated as an average % baseline of fEPSP slope for the first 10 min (1–10 min) or last 11 min (50–60 min) post TBS. The potentiation of the fEPSP slope was expressed as a percentage change relative to the pre-TBS baseline.

### In vivo multiphoton calcium imaging

The procedure for animal preparation followed the same protocol as described previously [3, 9]. Briefly, C57Bl/6 mice were placed in an induction box and anesthetized using isoflurane (~ 3–4%). Following induction, animals were transferred to a stereotaxic frame and heating plate (37–38 °C) and maintained using 1–1.5% isoflurane during surgical procedures, with respiration and pulse rate continuously monitored. The skin was first carefully excised and retracted, and a custom-made recording chamber/well affixed to the exposed skull. Subsequently, a small craniotomy (1 mm^2^, 2.5 mm posterior to bregma, 2.2 mm lateral to the midline) was performed and the exposed cortical tissue carefully aspirated to reveal the underlying hippocampus (CA1). The recording chamber was perfused with warmed Ringer’s solution (in nM): 125 NaCl, 4.5 KCl, 26 NaHCO_3_, 1.25 NaH_2_PO_4_, 2 CaCl_2_, 1 MgCl_2_ and 20 glucose, pH 7.4, 95% O_2_ and 5% CO_2_, and the hippocampus stained using Fluo-8®, AM (0.6 mM) (AAT Bioquest, Inc., Sunnyvale, CA, USA) via the multi-cell bolus loading injection technique [10]. Peptides (100 nM) were perfused into the recording chamber for bath application to the exposed CA1 region of the hippocampus (45–60 min wash in). In vivo imaging was conducted as described previously using a custom-built two-photon microscope consisting of a titanium:sapphire laser (coherent; λ=925 nm), resonant scanner, a Pockels cell laser modulator, and a water-immersion objective (Nikon; 40 × 0.8 numerical aperture) [3]. Images were acquired at a sampling rate of 30 Hz using custom-written LabVIEW routines and analyzed off-line in LabVIEW (National Instruments, Austin, TX, USA), Igor Pro (WaveMetrics, Inc., Lake Oswego, OR, USA), and MATLAB (The MathWorks, Natick, MA, USA). Regions of interest (ROIs) were manually defined around individual cell bodies, and time series of relative calcium fluorescence changes (ΔF/F) were extracted for each ROI. Significant changes in fluorescence were defined as ΔF/F calcium transients which exceeded background noise levels by > 3 standard deviations (SD), in accord with the analytical approach used in our previous publication studying Aη peptides using multiphoton calcium imaging [3] and for direct comparison with the current study. Animals were maintained at low levels of isoflurane anesthesia (~ 0.8%) throughout imaging procedures.

### Statistical analysis

Detailed statistics are presented in supplementary Tables S1, S2, S3, S4 and S5. Results are shown as mean ± S.E.M. Significant effects were inferred at *p*< 0.05.

For ex vivo and in vivo electrophysiology, statistical analyses were performed with Prism GraphPad 6.0 Software (GraphPad Software, La Jolla, CA, USA). “N” refers to the number of animals and “n” to the number of slices examined. For ex vivo electrophysiology data analysis, each peptide condition was plotted and analyzed against its own interleaved controls performed within the same experimental period. The normality of data distribution was verified with Shapiro-Wilk’s test. When normally distributed, an unpaired Student’s two-tailed t-test was used for comparison of two independent samples. When normality was not observed, a Mann-Whitney test was used for comparison of two independent samples. For comparison of more than 2 conditions, a one-way or two-way ANOVA was used followed by Dunnett’s test or Sidak’s tests for post hoc comparisons, as appropriate.

Statistical analyses of in vivo calcium imaging data were performed using MATLAB (The MathWorks, Natick, MA, USA). Following testing for normality using a one-sample Kolmogorov-Smirnov, the non-parametric Mann-Whitney U test was used to test for equality of population medians. For statistical testing of more than two groups, the nonparametric Kruskal-Wallis test was used with Tukey-Kramer correction for multiple comparisons. The experimenter was blinded to the synthetic peptide being administered and corresponding data only decoded at the end of experimentation.

## Results

### sAη–α and sAη–β impair LTP ex vivo within low nanomolar range

**Fig. 1:**
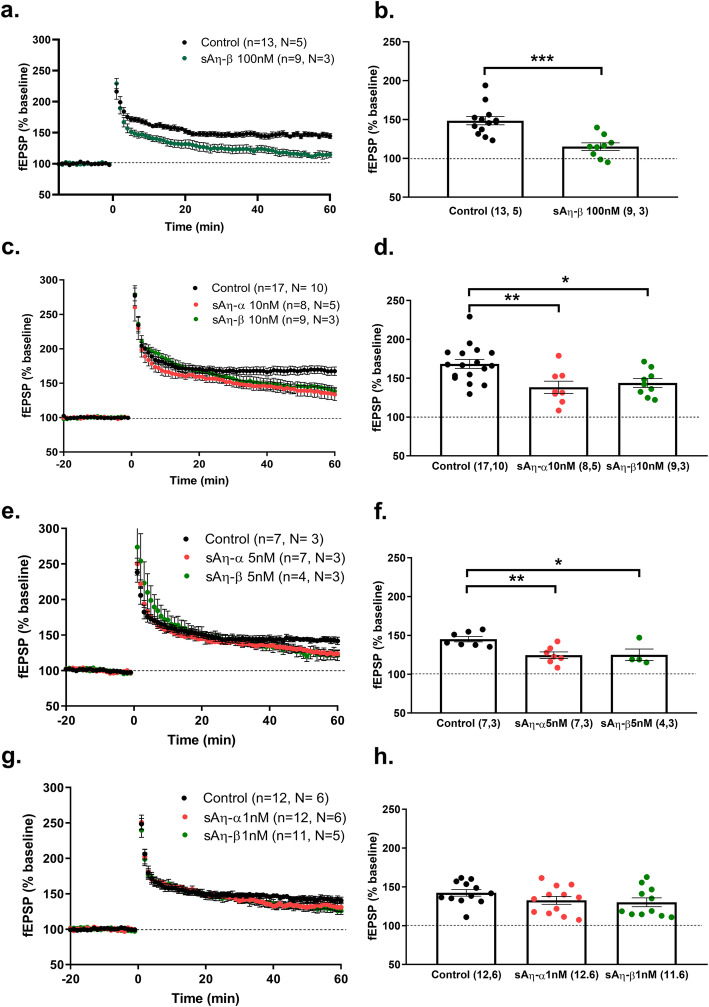
Synthetic Aη-α and Aη-β acutely inhibit LTP within the low nanomolar range. LTP was analyzed ex vivo at CA3-CA1 synapse in hippocampal slices of RjOrl:SWISS mice. **a**, **c**, **e**, and **g** Summary graphs of fEPSP slope (% baseline) pre- and post-LTP induction (time 0) in control (aCSF only) or in presence of **a** 100 nM sAη–β, **c** 10 nM sAη–α or sAη–β, **e** 5 nM sAη–α or sAη–β, and **g** 1 nM sAη–α or sAη–β, throughout the recording. **b**, **d**, **f**, and **h** Summary of fEPSP magnitude 45–60 min after LTP induction as fEPSP (% baseline) for data shown in **a**, **c**, **e**, and **g**, respectively (n= slices, N= mice), *p< 0.05, **p< 0.01, ***p< 0.001. Detailed statistics are shown in Supplementary Table S1

Previously, we reported an impairment of LTP at the CA3–CA1 synapse upon acute exposure of adult mouse hippocampal slices to synthetic Aη–α (sAη–α) at 100 nM [3]. We now report that synthetic Aη–β (sAη–β) also lowers LTP at 100 nM (Fig. 1a, b). The previously published and present data demonstrating that Aη peptides lower LTP were obtained in adult RjOrl:SWISS mice. We however confirmed that the peptide’s effect on LTP is independent of mouse strain, as 100 nM sAη-α also impaired LTP in hippocampal slices of C57Bl/6 mice (control: 158 ± 6,84, n= 17, N= 5; 10 nM sAη-α: 125.9 ± 8,28, n= 8, N= 2; p=0.01; Mann-Whitney test, see supplementary Table S1 for full statistics).

To exclude putative high-dose concentration-related toxicity, we now aimed to identify the minimal dose at which both sAη-α and sAη–β can affect LTP. We therefore tested 10, 5, and 1 nM doses. Application of both 10 nM and 5 nM was sufficient to significantly reduce LTP response compared to respective control conditions (Fig. 1c–f), an effect which did not persist at 1 nM (Fig. 1g, h). We conclude that these peptides are active within the low nanomolar range.

sAη–α’s effect on LTP could be correlated to a modulation of short-term pre-synaptic plasticity of neurotransmitter release or basic synaptic transmission in hippocampal slices. We measured paired-pulse ratios (PPRs), a commonly used indicator of short-term presynaptic plasticity. sAη–α (10 nM) did not perturb the PPRs, as we observed a similar facilitation of synaptic release in both conditions at 100 ms ISI that gradually decreased with increasing ISI (Supplementary Figure S1a). This result indicates that sAη–α does not modulate short-term pre-synaptic plasticity mechanisms. We also observed that sAη–α (10 nM) did not modulate baseline synaptic transmission (Supplementary Figure S1b-c).

### Ten nanomolar of recombinant Aη–β impairs LTP ex vivo

**Fig. 2:**
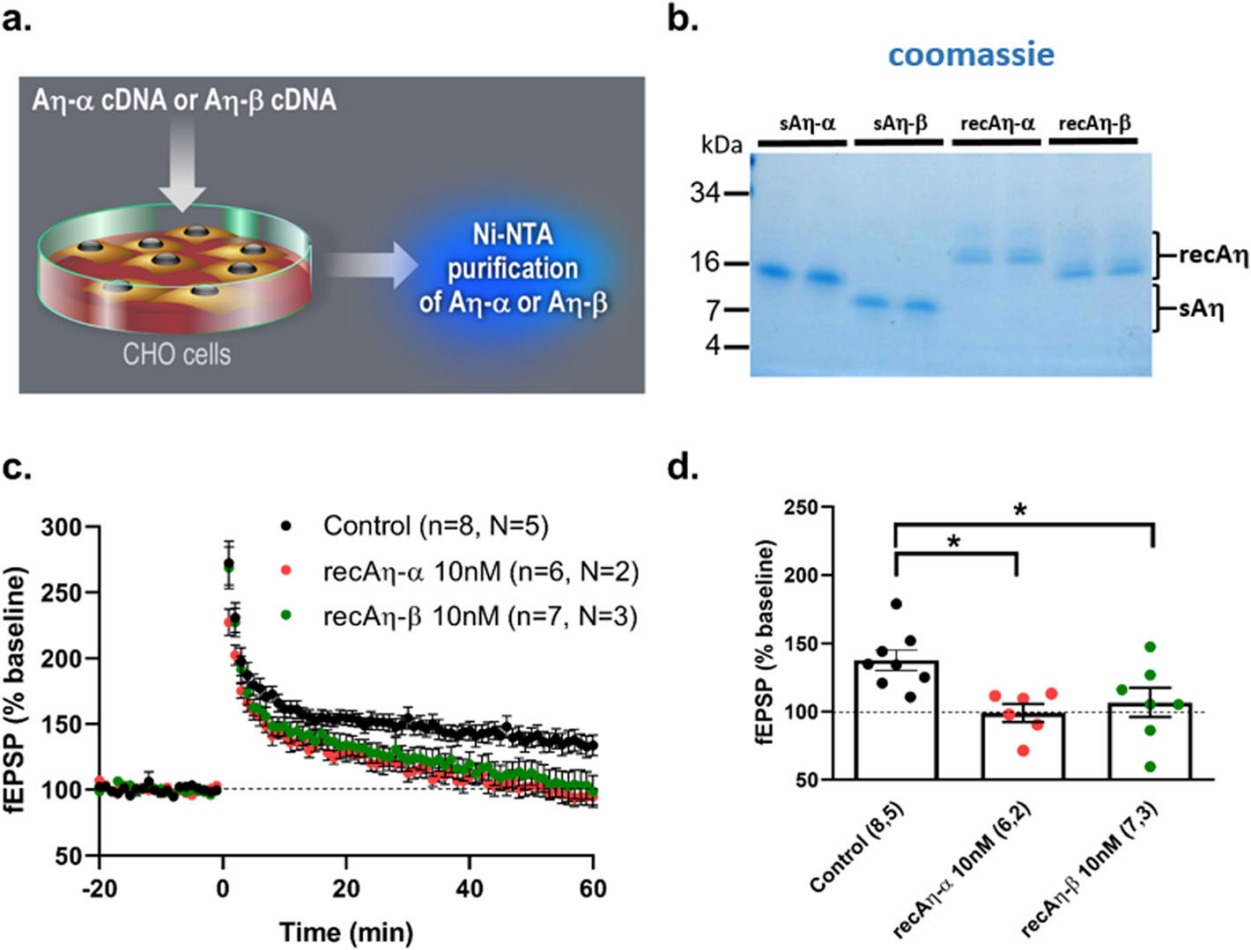
Ten nanomolar of soluble recombinant Aη-α and Aη-β lower LTP*.* **a** Diagram explaining the production of soluble HIS-tagged recAη-α and recAη-β samples used in **c** and **d** (see the “Methods” section for details of Ni-NTA purification and sample quantification). **b** Commassie stain of 1 μg of Aη peptides. Synthetic Aη peptides or recombinant CHO cell-expressed glycosylated purified Aη peptides were separated in SDS-PAGE and stained with GelCode Blue. **c**, **d** LTP was analyzed ex vivo at CA3-CA1 synapse in hippocampal slices of RjOrl:SWISS mice. Summary graphs of **c** fEPSP slope (% baseline) pre- and post-LTP induction (time 0) and **d** fEPSP magnitude 45–60 min after LTP induction in control (aCSF only) or in presence of 10 nM recAη–α or recAη–β throughout the recording (n= slices, N= mice), *p< 0.05. Detailed statistics are shown in Supplementary Table S2

We previously reported that recombinant forms of these peptides, produced from CHO cell (Fig. 2a), differently impacted LTP ex vivo with recAη-α but not rec Aη-β lowering LTP [3]. Of note, in this first study, we had not carefully assessed the concentration of the recombinant peptides that were applied during LTP analysis, resorting only to equal dilution (1/15) of purified samples. Since we realized later that the synthetic form of Aη-β lowers LTP (Fig. 1), a result in direct contradiction with this previous finding, we tested again the activity of recombinant Aη-α and Aη-β after careful assessment of concentration. As exemplified in Fig. 2b, in the present study, we now quantified new preparations of recombinant Aη-α and Aη-β peptides by nanodrop measurements and verified quantities by comparison to known concentrations of synthetic peptides are shown in a Coomassie blue staining. These recombinant purified peptides are composed of the main protein band and additional less abundant bands with slightly higher molecular weights (Fig. 2b). As these additional bands are not observed with the synthetic peptides, they are likely to represent post-translation modifications, such as O-glycosylation, which was shown for this stretch of amino acids as discussed by others [11]. When applying 10 nM of these recombinant peptides on hippocampal slices, we report here that both 10 nM recAη-α and recAη-β, like their synthetic counterparts, significantly lower LTP (Fig. 2c, d).

### N-terminus sequence of Aη is necessary and sufficient for LTP impairment

**Fig. 3:**
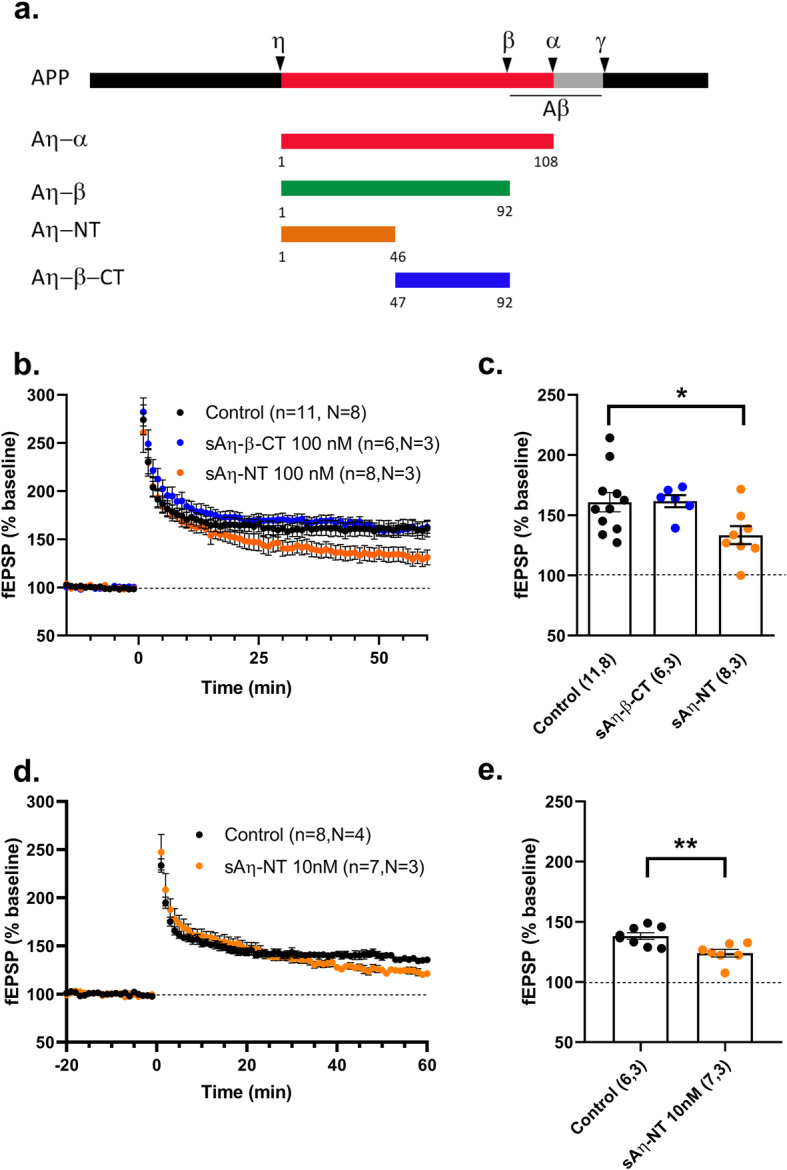
N-terminal of Aη is necessary and sufficient for LTP impairment. **a** Diagram showing APP processing (secretases cleavage sites are shown) and boundaries of shorter synthetic peptides used to identify the active site. **b**–**e** LTP was analyzed ex vivo at CA3-CA1 synapse in hippocampal slices of RjOrl:SWISS mice. Summary graphs of **b**, **d** fEPSP slope (% baseline) pre- and post-LTP induction (time 0) and **c**, **e** fEPSP magnitude 45–60 min after LTP induction in control (aCSF only) or in presence of **b**, **c** 100 nM sAη–NT or sAη–β-CT or **d**, **e** of 10 nM sAη–NT, throughout the recording (n= slices, N= mice), *p< 0.05. Detailed statistics are shown in Supplementary Table S3

Next, we aimed at identifying the region in Aη that mediates the effect on LTP. We synthesized two other peptides (46 amino acids each) representing the N-terminal and C-terminal portions of sAη–β, that we termed sAη–NT and sAη–β–CT, respectively (Fig. 3a). We first tested their effect on LTP at 100 nM. Whereas sAη–NT, comprising the N-terminal stretch of amino acids present in both sAη–α and sAη–β, lowered LTP, and sAη–β–CT failed to recapitulate this effect (Fig. 3b, c). We also tested sAη–NT at 10 nM and report that, like Aη–α and sAη–β, sAη–NT impaired LTP at this lower concentration (Fig. 3d, e). Taken together, these findings indicate that the N-terminal part of Aη is necessary and sufficient for LTP impairment.

### sAη peptides induce hippocampal neuronal hypoactivity in vivo

**Fig. 4:**
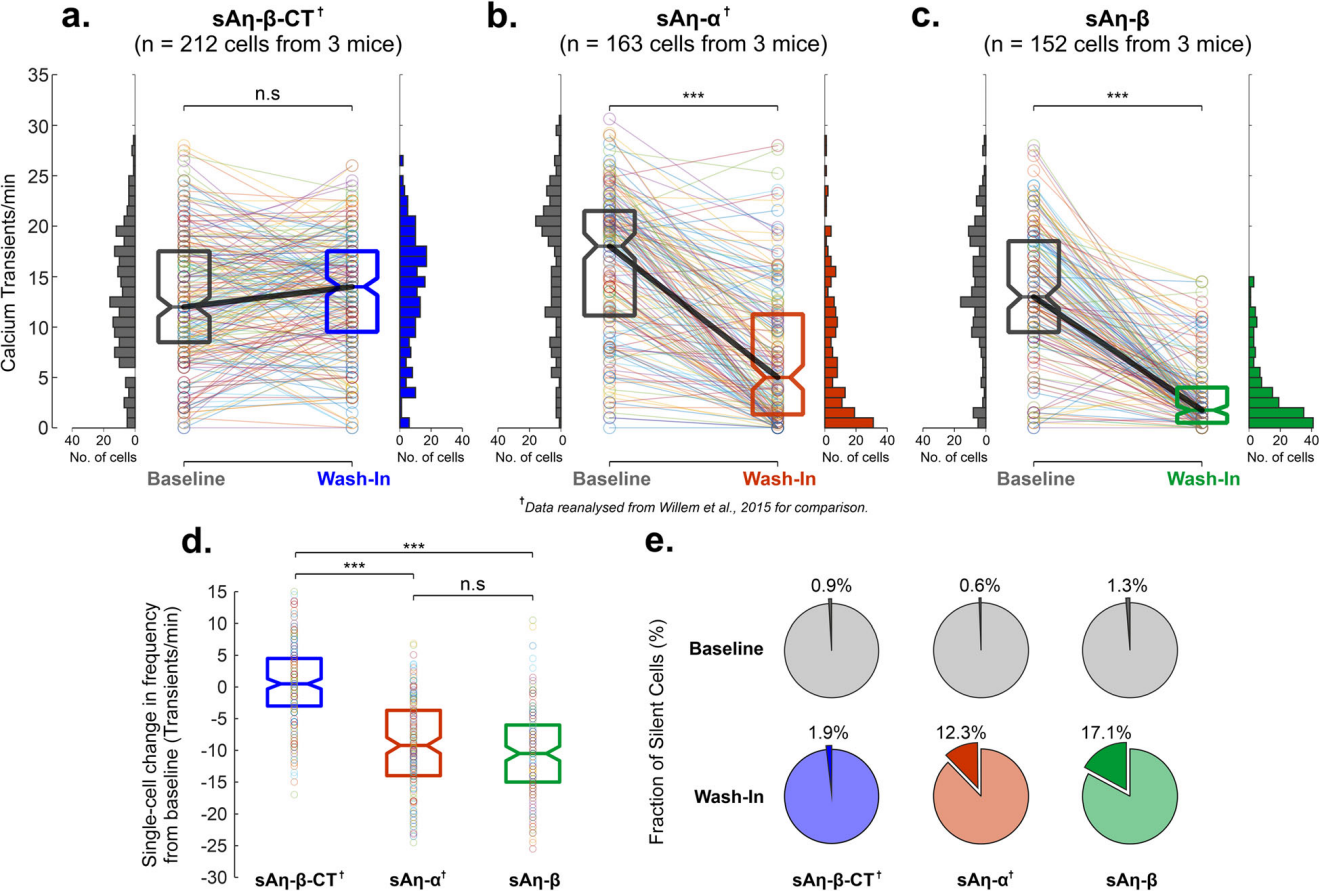
Synthetic Aη-α and Aη-β acutely modulate neuronal activity in vivo. **a** The median frequency of calcium transients in the CA1 region of hippocampi of C57Bl/6 mice did not differ significantly (n.s) from baseline following superfusion (wash-in, blue) of sAη–β–CT (control peptide). Note the high degree of similarity in distribution of calcium transient frequencies as denoted by the upright histograms. The frequency of calcium transients for each individual neuron before (baseline) and after superfusion (wash-in) of sAη–β–CT is overlaid. **b** Superfusion of sAη–α (wash-in, red) induced a significant decrease in median calcium transient frequency and a positive skew in the distribution of calcium transient frequencies towards hypoactivity. The frequency of calcium transients for each individual neuron before (baseline) and after superfusion (wash-in) of sAη–α is overlaid. **c** Superfusion of sAη–β (wash-in, green) also induced a significant decrease in median calcium transient frequency and a positive skew in the distribution of calcium transient frequencies towards hypoactivity. The frequency of calcium transients for each individual neuron before (baseline) and after superfusion (wash-in) of sAη–β is overlaid. **d** Across all individual cells studied, the median change in calcium transient frequency following superfusion of sAη–α (red) and sAη–β (green) was not statistically different (n.s), but were significantly lower relative to the null change associated with sAη–β–CT (blue). The change in calcium transient frequency for each individual neuron after superfusion (wash-in) of each peptide is overlaid. **e** While the proportion of silent neurons (i.e. showing absence of calcium transients) were small during baseline conditions (grey charts), and following sAη–β–CT superfusion (wash-in, blue), superfusion of sAη–α (wash-in, red) and sAη–β (wash-in, green) was associated with a dramatic increase in the number of inactive cells. For each boxplot, the central line denotes the median with bottom and top demarcations indicating the 25th and 75th percentiles, respectively. Data for sAη–α and sAη-β-CT were previously published [3] and presented and reanalyzed in this figure for comparison purposes (denoted as^†^). ***p< 0.001. Detailed statistics are shown in Supplementary Table S4

We previously reported that sAη–α (100 nM) suppressed neuronal activity in vivo by using multiphoton calcium imaging at single-cell resolution [3]. Here we performed the same experiment using sAη–β, with sAη–β–CT as the control peptide, at the dose of 100 nM as used in the previous study [3]. This allowed us to compare the effects of sAη–α and sAη–β versus sAη–β–CT with reanalysis of our previously published data [3]. We found that sAη–α and sAη–β superfusion (“wash-in”) resulted in a significant reduction in neuronal activity, as measured by the spontaneous frequency of calcium transients in CA1 from baseline, with no significant effects induced by the N-terminally truncated control peptide sAη–β–CT (Fig. 4a–d). In contrast to sAη–β–CT, both sAη–α and sAη–β were associated with an increase in the number of silent neurons (Fig. 4e), indicating that both synthetic Aη peptides induce profound neuronal hypoactivity in vivo.

### sAη–α also lowers LTP in vivo

**Fig. 5:**
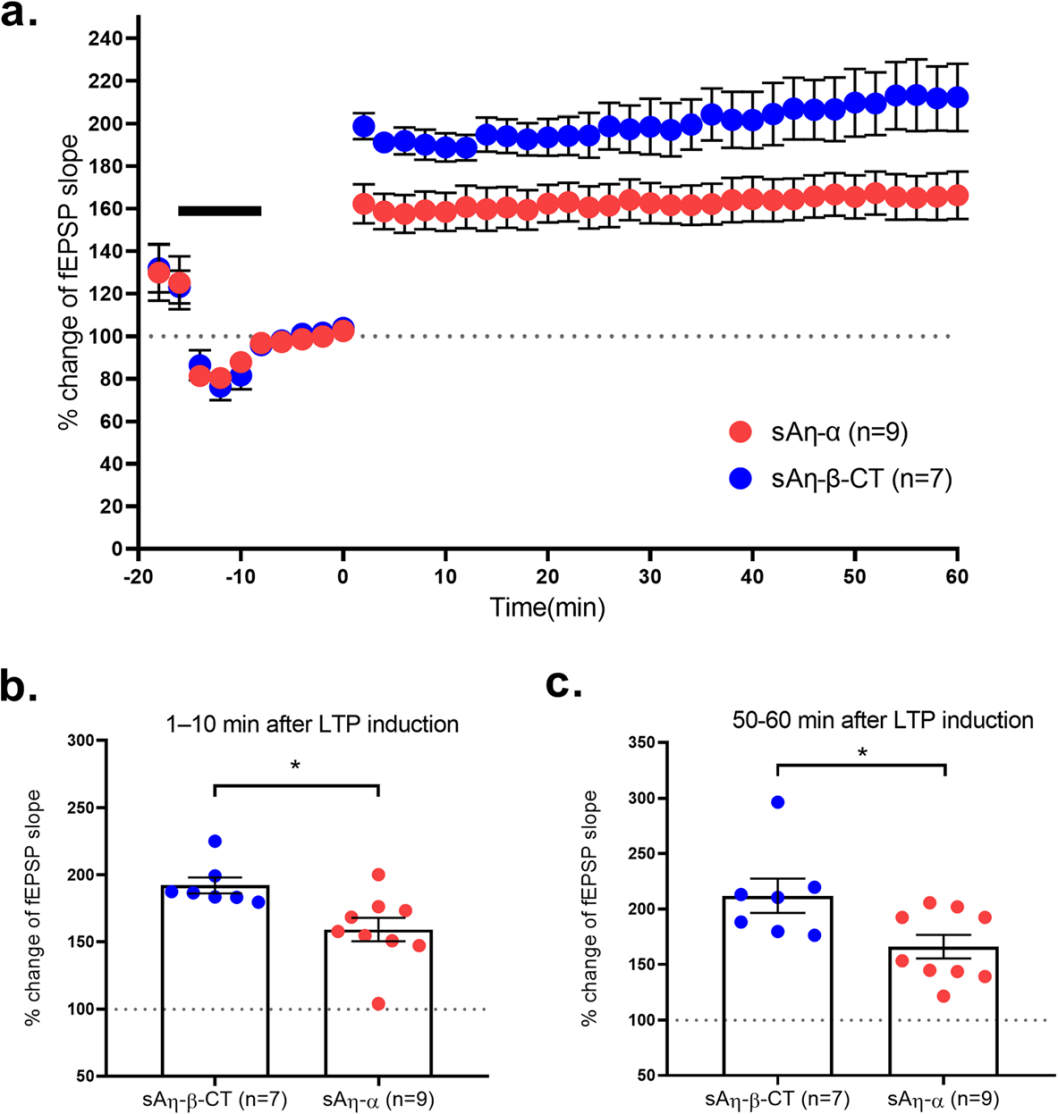
sAη–α acutely lowers LTP in vivo. **a** LTP was measured in vivo in the dentate gyrus of Sprague-Dawley rats. Summary graphs of fEPSP slope (% baseline) pre- and post-LTP induction (time 0) upon 10 min of intra-hippocampal injection of 1 μM of control peptide (sAη–β–CT) or sAη–α. Injection time is shown by the black bar. Note a transient decline and partial recovery of baseline responses upon the injection and a subsequent LTP induction. **b** Summary of fEPSP magnitude 1–10 min after LTP induction as fEPSP (% baseline) for data shown in **a**. **c** Summary of fEPSP magnitude 50–60 min after LTP induction as fEPSP (% baseline) for data shown in **a**. All recordings were done in vivo in Sprague Dawley rats with n= number of rats. *p< 0.05 Detailed statistics are shown in Supplementary Table S5

Finally, we tested if sAη–α, representing the most abundant form of Aη in vivo [3], also impairs long-term synaptic plasticity in the intact circuitry of live animals, by measuring its impact on LTP in the hippocampus in vivo. For this purpose, 1 μM sAη–α was acutely applied into the hippocampal dentate gyrus of urethane-anesthetized rats. This higher dose of 1 μM was chosen to increase the likelihood of detecting an effect. Indeed, unlike for ex vivo LTP and in vivo calcium imaging for which the peptides under analysis were constantly recirculated during the experiment, here we acutely applied the peptide only once at the beginning of the recording with the possibility of diffusion, degradation, or uptake of the peptide in vivo during the rest of the recording. As a control peptide, we used sAη–β–CT (1 μM) that did not impact LTP ex vivo (Fig. 3) nor neuronal activity in vivo (Fig. 4). LTP was induced by TBS of perforant path synaptic inputs in the dentate gyrus [12, 13]. TBS was applied after a typical transient decline and recovery of baseline fEPSP responses upon the brief injection of 1 μl fluid containing sAη–α or control peptide (see Fig. 5a and the “Methods” section). Following TBS, significant LTP of the fEPSPs was observed in both groups of rats. However, sAη–α efficiently lowered LTP when compared to control peptide during both the induction (Fig. 5b) and maintenance (Fig. 5c) of LTP. These data demonstrate that sAη–α is also able to reduce LTP in the living brain.

## Discussion

In this report, we provide important novel insights into the activity of Aη peptides at excitatory hippocampal synapses. Notably, we demonstrate that both the long and short forms of Aη are active within the low nanomolar range that is strongly indicative of physiological relevance. Specifically, we report that the recombinant form of the Aη-β lowers LTP as does Aη-α. We provide confirmation of the impact of sAη–α on LTP in the intact brain. We show that, like sAη-α, sAη-β also induces neuronal hypoactivity in vivo. Together these data suggest that both peptides share a similar bioactive profile. Finally, we provide novel evidence that the active region of Aη is located within its N-terminus sequence.

The Aη-dependent impairment of LTP is reminiscent of the activity of Aβ, in its dimer or oligomeric forms, at these same synapses [14–16]. Aη–α overlaps with the N-terminus of Aβ by 16 amino acids and it thus may be tempting to speculate that Aη–α-mediated effects on synaptic plasticity reflect a common active site. Yet, our data do not support this interpretation for several reasons. First, we found a similar LTP impairment with sAη–β, which does not harbor these overlapping 16 amino acids. Second, the likelihood of the active site lying in N-terminal portion of the Aη sequence was supported by our observation that, while both sAη–NT and sAη–β–CT are present in Aη peptides but not in Aβ, only sAη–NT lowered LTP ex vivo*.* Finally, the effects of Aβ on LTP are only observed with aggregated Aβ [16, 17], in contrast to the monomeric activity of Aη peptides, which mediate these effects since these peptides do not oligomerize [3].

We show here that these Aη peptides acutely modulate glutamatergic signaling. Our data point towards an action of the peptides at the post-synapse which preferentially targets mechanisms that control LTP and spontaneous neuronal activity in vivo, since baseline synaptic transmission and pre-synaptic short-term plasticity were not affected by Aη-α. Future studies should focus on the identification of the exact Aη-dependent molecular interactions with post-synaptic mechanisms. Perturbations in synaptic glutamatergic signaling are a hallmark of AD leading to excitatory synapse failure [5]. Yet, there is currently very little information on the relevance of the Aη peptides in AD. We previously showed that the C-terminal fragment of the η-secretase pathway (CTF-η) accumulates in the halo of Aβ plaques in an APPPS1 mouse model of AD and that dystrophic neurites of hippocampi of human AD patients are positive for Aη-epitope antibodies [3]. In light of the new data we present here, future analysis of synapse dysfunction in the context of AD should take into consideration the possibility that Aη peptide-related mechanisms also contribute to AD-linked synapse failure, especially in the hippocampus for which we are providing functional evidence.

Finally, our finding that Aη peptides impact LTP and neuronal excitability within the low nanomolar range emphasizes that care should be taken when designing therapeutic strategies for AD, such as when increasing Aη–α brain levels through BACE-1 inhibition [3]. Furthermore, since BACE-1 inhibition would also be expected to increase the generation of soluble APP species (sAPPα), recently reported to reduce neuronal activity in vivo [18], the resulting suppressive effect of both peptide species on neuronal activity might well be pronounced and may partly explain observations of acute adverse cognitive outcomes in recent BACE-1 targeting clinical trials [19, 20].

## Limitations

Although the physiological concentrations of Aη peptides within the brain remain unknown, several lines of evidence suggest that our findings may reflect the physiological endogenous activity of these peptides in vivo. Previous data has indicated that the range of Aβ concentrations in rodents and humans to be within the high picomolar range, with CSF levels of Aβ_1-40_ in healthy humans estimated to be around 1.5 nM [14, 21–23]. We have also previously estimated endogenous Aη–α levels to be five-fold higher in CSF than Aβ [3], suggesting that Aη CSF levels might be expressed in the region of 7.5 nM. The range of 5–10 nM, for which we observed an impact of Aη on synapses, seems therefore within the estimated realm of the endogenous concentration of this peptide. We currently have no information on how endogenous Aη concentrations fluctuate with neuronal activity, but this has already been observed for Aβ in vivo [24–27] and it will thus be important to investigate this possibility with respect to these new peptides. It is conceivable that our observations reflect a novel form of physiological regulation of post-synaptic plasticity mechanisms by these peptides as a consequence of expression level fluctuations due to neuron activity.

Intriguingly, our finding that the recombinant form of Aη-β lowers LTP is at variance with that found in our previous study [3], despite the derivation procedure of recombinant Aη-β being essentially similar as described here. Since we did not quantify the precise concentration of recombinant peptide in our earlier study, it is possible that the concentration applied previously failed to reach the limit of detection for an effect on LTP. Alternatively, it is possible that factors, such as variable O-glycosylation levels, purity, or degradation of samples, may have confounded some of our previous results with the Aη-β recombinant peptide, which were mitigated in the current study through additional quality control steps until application.

Our work specifically assesses the acute effect of adding Aη peptides on neurons. We currently hold no information on how the potential accumulation of these peptides in the brain could chronically modulate neuron function, including LTP. In a related line of work, we are beginning to assess how chronic over-expression of Aη-α modulates brain information processing using a newly generated mouse model. Also, the necessity of these peptides in physiological conditions or their contribution to AD pathogenesis is currently unknown. To assess this, one would have to reduce the endogenous levels of the Aη peptides in physiological or pathological settings. In our previous work, we reported that MT5-MMP displays η-secretase activity [3]. One study further showed that the absence of MT5-MMP in the 5xFAD mouse model of AD decreased amyloid burden, preserved LTP, and improved cognitive performance, suggesting that this proteinase is implicated in AD pathogenesis [4]. It is difficult to specifically impact η-secretase-dependent APP cleavage pharmacologically with secretase inhibitors as these enzymes, including MT5-MMP, process other substrates beside APP. To more specifically address the physiological relevance of APP peptides generated by the η-secretase pathway, one could resort to genetic ablation of the secretase site on APP thus preventing endogenous processing by η-secretase. To date, this type of approach has not been reported and little information is available as to the necessity of these peptides in the brain. Once we better identify the role of Aη peptides in brain information processing in physiopathological settings, one could envisage the design and use of targeted pharmacological agents to modulate their levels to treat Aη-related brain alterations.

## Conclusions

In conclusion, we demonstrate that both Aη peptides acutely regulate neuronal mechanisms ex vivo and in vivo and could thus represent important endogenous modulators of synapse communication. Our findings provide further evidence that, beyond Aβ, APP cleavage products contribute to a rich array of effects on neuronal function [28], which delicately maintains neuronal network homeostasis and may be uniquely susceptible to perturbation.

## Supplementary Information

**Additional file 1: Figure S1.** Acute application of sAƞ-α (10 nM) does not perturb short-term pre-synaptic plasticity nor basal excitatory synaptic transmission. **Table S1.** Statistics of Fig. 1 and S1 and C57Bl/6 ex vivo LTP data. **Table S2.** Statistics of Fig. 2. **Table S3.** Statistics of Fig. 3. **Table S4.** Statistics of Fig. 4. **Table S5.** Statistics of Fig. 5.

## Data Availability

The datasets used and/or analyzed during the current study are available from the corresponding author on reasonable request.

## Abbreviations

aCSF: Artificial cerebrospinal fluid
AD: Alzheimer’s disease
APP: Amyloid precursor protein
BACE1: β-secretase-1 cleavage site
CSF: Cerebrospinal fluid
DMSO: Dimethyl sulfoxide
ECL: Enhanced chemiluminescence
fEPSPs: Field excitatory post-synaptic potentials
HEPES: 4-(2-hydroxyethyl)-1-piperazineethanesulfonic acid
HFS: High-frequency stimulation
ISI: Inter-stimulus interval
LTP: Long-term potentiation
MT1/5-MMP: Membrane types 1 and 5 matrix metalloproteinases
PBS: Phosphate-buffered saline
Rec: Recombinant
ROIs: Regions of interest
s: Synthetic
SD: Standard deviation
SEM: Standard error of the mean
TBS: Theta-burst stimulation

## References

[1] van der Kant R, Goldstein LSB (2015). Cellular functions of the amyloid precursor protein from development to dementia. Dev Cell.

[2] García-González L, Pilat D, Baranger K, Rivera S (2019). Emerging alternative proteinases in APP metabolism and Alzheimer’s disease pathogenesis: a focus on MT1-MMP and MT5-MMP. Front Aging Neurosci.

[3] Willem M, Tahirovic S, Busche MA, Ovsepian SV, Chafai M, Kootar S, Hornburg D, Evans LDB, Moore S, Daria A, Hampel H, Müller V, Giudici C, Nuscher B, Wenninger-Weinzierl A, Kremmer E, Heneka MT, Thal DR, Giedraitis V, Lannfelt L, Müller U, Livesey FJ, Meissner F, Herms J, Konnerth A, Marie H, Haass C (2015). eta-Secretase processing of APP inhibits neuronal activity in the hippocampus. Nature.

[4] Baranger K, Marchalant Y, Bonnet AE, Crouzin N, Carrete A, Paumier JM, Py NA, Bernard A, Bauer C, Charrat E, Moschke K, Seiki M, Vignes M, Lichtenthaler SF, Checler F, Khrestchatisky M, Rivera S (2016). MT5-MMP is a new pro-amyloidogenic proteinase that promotes amyloid pathology and cognitive decline in a transgenic mouse model of Alzheimer’s disease. Cell Mol Life Sci.

[5] Selkoe DJ (2002). Alzheimer’s disease is a synaptic failure. Science.

[6] De Strooper B, Karran E (2016). The cellular phase of Alzheimer’s disease. Cell.

[7] Frere S, Slutsky I (2018). Alzheimer’s disease: from firing instability to homeostasis network collapse. Neuron.

[8] Taylor CJ, Ireland DR, Ballagh I, Bourne K, Marechal NM, Turner PR, Bilkey DK, Tate WP, Abraham WC (2008). Endogenous secreted amyloid precursor protein-alpha regulates hippocampal NMDA receptor function, long-term potentiation and spatial memory. Neurobiol Dis.

[9] Busche MA (2012). Critical role of soluble amyloid-beta for early hippocampal hyperactivity in a mouse model of Alzheimer’s disease. Proc Natl Acad Sci U S A.

[10] Stosiek C, Garaschuk O, Holthoff K, Konnerth A (2003). In vivo two-photon calcium imaging of neuronal networks. Proc Natl Acad Sci U S A.

[11] Akasaka-Manya K, Manya H. The role of APP O-glycosylation in Alzheimer’s disease. Biomolecules. 2020;10(11). 10.3390/biom10111569. 10.3390/biom10111569 PMC7699271 33218200

[12] Jedlicka P, Vnencak M, Krueger DD, Jungenitz T, Brose N, Schwarzacher SW (2015). Neuroligin-1 regulates excitatory synaptic transmission, LTP and EPSP-spike coupling in the dentate gyrus in vivo. Brain Struct Funct.

[13] Vnencak M, Schölvinck ML, Schwarzacher SW, Deller T, Willem M, Jedlicka P (2019). Lack of β-amyloid cleaving enzyme-1 (BACE1) impairs long-term synaptic plasticity but enhances granule cell excitability and oscillatory activity in the dentate gyrus in vivo. Brain Struct Funct.

[14] Puzzo D, Privitera L, Leznik E, Fa M, Staniszewski A, Palmeri A, Arancio O (2008). Picomolar amyloid-beta positively modulates synaptic plasticity and memory in hippocampus. J Neurosci.

[15] Townsend M, Shankar GM, Mehta T, Walsh DM, Selkoe DJ (2006). Effects of secreted oligomers of amyloid beta-protein on hippocampal synaptic plasticity: a potent role for trimers. J Physiol.

[16] Shankar GM, Li S, Mehta TH, Garcia-Munoz A, Shepardson NE, Smith I, Brett FM, Farrell MA, Rowan MJ, Lemere CA, Regan CM, Walsh DM, Sabatini BL, Selkoe DJ (2008). Amyloid-beta protein dimers isolated directly from Alzheimer’s brains impair synaptic plasticity and memory. Nat Med.

[17] Li S, Hong S, Shepardson NE, Walsh DM, Shankar GM, Selkoe D (2009). Soluble oligomers of amyloid Beta protein facilitate hippocampal long-term depression by disrupting neuronal glutamate uptake. Neuron.

[18] Rice HC, de Malmazet D, Schreurs A, Frere S, van Molle I, Volkov AN, Creemers E, Vertkin I, Nys J, Ranaivoson FM, Comoletti D, Savas JN, Remaut H, Balschun D, Wierda KD, Slutsky I, Farrow K, de Strooper B, de Wit J (2019). Secreted amyloid-β precursor protein functions as a GABABR1a ligand to modulate synaptic transmission. Science.

[19] Egan MF, Kost J, Tariot PN, Aisen PS, Cummings JL, Vellas B, Sur C, Mukai Y, Voss T, Furtek C, Mahoney E, Harper Mozley L, Vandenberghe R, Mo Y, Michelson D (2018). Randomized trial of verubecestat for mild-to-moderate Alzheimer’s disease. N Engl J Med.

[20] Egan MF, Kost J, Voss T, Mukai Y, Aisen PS, Cummings JL, Tariot PN, Vellas B, van Dyck CH, Boada M, Zhang Y, Li W, Furtek C, Mahoney E, Harper Mozley L, Mo Y, Sur C, Michelson D (2019). Randomized trial of verubecestat for prodromal Alzheimer’s disease. N Engl J Med.

[21] Schmidt SD, Nixon RA, Mathews PM (2005). ELISA method for measurement of amyloid-beta levels. Methods Mol Biol.

[22] Giedraitis V, Sundelöf J, Irizarry MC, Gårevik N, Hyman BT, Wahlund LO, Ingelsson M, Lannfelt L (2007). The normal equilibrium between CSF and plasma amyloid beta levels is disrupted in Alzheimer’s disease. Neurosci Lett.

[23] Puzzo D, Arancio O (2013). Amyloid-β peptide: Dr. Jekyll or Mr. Hyde?. J Alzheimers Dis.

[24] Nitsch RM, Farber SA, Growdon JH, Wurtman RJ (1993). Release of amyloid beta-protein precursor derivatives by electrical depolarization of rat hippocampal slices. Proc Natl Acad Sci U S A.

[25] Cirrito JR, Yamada KA, Finn MB, Sloviter RS, Bales KR, May PC, Schoepp DD, Paul SM, Mennerick S, Holtzman DM (2005). Synaptic activity regulates interstitial fluid amyloid-beta levels in vivo. Neuron.

[26] Cirrito JR, Kang JE, Lee J, Stewart FR, Verges DK, Silverio LM, Bu G, Mennerick S, Holtzman DM (2008). Endocytosis is required for synaptic activity-dependent release of amyloid-beta in vivo. Neuron.

[27] Kang JE, Lim MM, Bateman RJ, Lee JJ, Smyth LP, Cirrito JR, Fujiki N, Nishino S, Holtzman DM (2009). Amyloid-beta dynamics are regulated by orexin and the sleep-wake cycle. Science.

[28] Harris SS, Wolf F, De Strooper B, Busche MA (2020). Tipping the scales: peptide-dependent dysregulation of neural circuit dynamics in Alzheimer’s disease. Neuron.

