# Supplementary material for: Aη-α and Aη-β peptides impair LTP ex vivo within the low nanomolar range and impact neuronal activity in vivo
Source: Alzheimers Res Ther. 2021 Jul 8;13:125. doi: 10.1186/s13195-021-00860-1 (PMC8268417; doi:10.1186/s13195-021-00860-1)
Supplement: Supplementary file 1 — Additional file 1: Figure S1. Acute application of sAƞ-α (10 nM) does not perturb short-term pre-synaptic plasticity nor basal excitatory synaptic transmission. Table S1. Statistics of Fig. 1 and S1 and C57Bl/6 ex vivo LTP data. Table S2. Statistics of Fig. 2. Table S3. Statistics of Fig. 3. Table S4. Statistics of Fig. 4. Table S5. Statistics of Fig. 5. [file 13195_2021_860_MOESM1_ESM.pdf]

**Aη−α and Aη−β peptides impair LTP *ex vivo* within the low nanomolar range and impact neuronal activity *in vivo*.**

Maria Mensch et al.

SUPPLEMENTARY MATERIAL

Supplementary Figure S1.
Supplementary Tables S1-5.

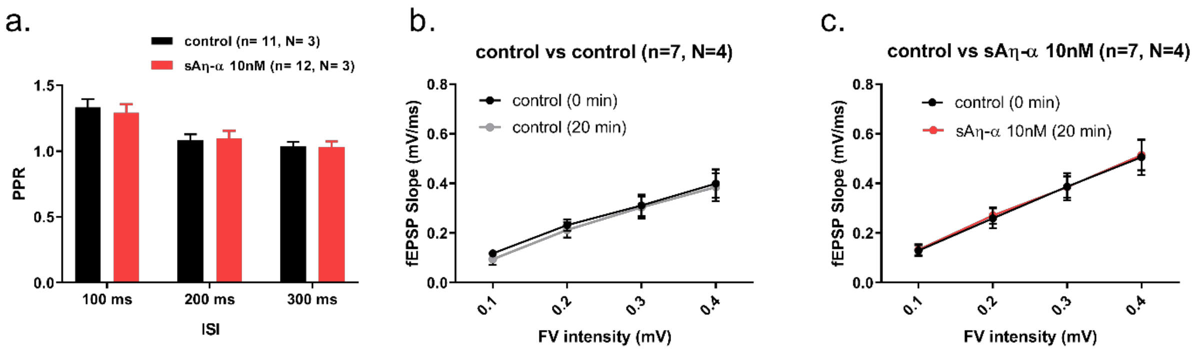


**Supplementary Figure S1. Acute application of sAη-α (10nM) does not perturb short-term pre-synaptic plasticity nor basal excitatory synaptic transmission.**

**a)** PPRs (fEPSP2 slope/fEPSP1 slope) were similar at all ISIs examined (100ms, 200ms, 300ms) in control condition (aCSF only) and in presence of 10nM sAη-α throughout recording. ISI: inter-stimulus interval.

**b)** Synapse input–output curves (fEPSP slopes versus increasing afferent FV amplitudes) were similar at time 0 and then after 20 minutes in control condition (aCSF only).

**c)** Synapse input– output curves (fEPSP slopes versus increasing afferent FV amplitudes) were similar at time 0 in control condition (aCSF only) and then after 20 minutes of incubation with sAη-α (10nM).

(n=slices, N=mice). Detailed statistics are shown in Supplementary Table S1.

## Table S1 - Statistics of Figure 1 and S1 and C57Bl/6 ex vivo LTP data

| Mouse model and genetic background | Experiment | | # mice | # slice | Measurement | Treatment | Average | S.E.M. | Units | Statistical Test | p value | F/t/z/R ETC value | Comparison | post-hoc test | p value | q(DF) value | Figure |
|---|---|---|---|---|---|---|---|---|---|---|---|---|---|---|---|---|---|
| RjOrl:SWISS | **LTP - 100 nM sAη–β** | | 5 | 13 | last 15 minutes | _ | 148,50 | 5,33 | % | Student t-test | **p=0,0003** | t=4,406 df=20 | - | - | - | - | **1b** |
| | | | 3 | 9 | | sAη-β | 115,10 | 4,79 | % | | | | | | | | |
| | **LTP - 10 nM sAη–α and sAη-β** | | 10 | 17 | last 15 minutes | _ | 168,40 | 5,77 | % | One-way ANOVA | **p=0,0039** | F(2,31)=6,670 | control vs sAη-α | Dunnett's test | **0,0061** | q(31)=3,203 | **1d** |
| | | | 5 | 8 | | sAη-α | 138,30 | 7,87 | % | | | | control vs sAη-β | | **0,0205** | q(31)=2,721 | |
| | | | 3 | 9 | | sAη-β | 143,80 | 5,66 | % | | | | | | | | |
| | **LTP - 5 nM sAη–α and sAPP-β** | | 3 | 7 | last 15 minutes | _ | 145,10 | 3,45 | % | One-way ANOVA | **p=0,0072** | F(2,15)=6,987 | control vs sAη-α | Dunnett's test | **0,0074** | q(15)=3,412 | **1f** |
| | | | 3 | 7 | | sAη-α | 124,50 | 4,23 | % | | | | control vs sAη-β | | **0,0235** | q(15)=2,836 | |
| | | | 3 | 4 | | sAη-β | 125,00 | 7,40 | % | | | | | | | | |
| | **LTP - 1 nM sAη–α and sAPP-β** | | 6 | 12 | last 15 minutes | _ | 142,30 | 4,24 | % | One-way ANOVA | p=0,2006 | f(2,32)=1,657 | control vs sAη-α | Dunnett's test | 0,2935 | q(32)=1,389 | **1h** |
| | | | 6 | 12 | | sAη-α | 132,60 | 5,11 | % | | | | control vs sAη-β | | 0,171 | q(32)=1,706 | |
| | | | 6 | 11 | | sAη-β | 130,10 | 5,66 | % | | | | | | | | |
| C57Bl/6 | **LTP - 100 nM sAη–α** | | 5 | 17 | last 15 minutes | _ | 158,00 | 6,84 | % | Mann Whitney Test | **p=0,0109** | U=25 | - | - | - | - | **-** |
| | | | 2 | 8 | | sAη-α | 125,90 | 8,28 | % | | | | | | | | |
| RjOrl:SWISS | **PPR 10nM sAη–α** | **100 ms** | 3 | 11 | EPSP slope | _ | 1,33 | 0,06 | % | Two-way ANOVA | interaction | interaction | Sidak's | Control vs sAη-α | p=0,9908 | t(84)= 0,3998 | **S1a** |
| | | | 3 | 12 | | sAη-α | 1,29 | 0,06 | % | | p=0,8842 | F (2, 63) = 0,1233 | | | | | |
| | | **200 ms** | 3 | 11 | | _ | 1,09 | 0,04 | % | | time | time | | Control vs sAη-α | p=0,9999 | t(84)= 0,1266 | |
| | | | 3 | 12 | | sAη-α | 1,10 | 0,06 | % | | **p<0,0001** | F (2, 63) = 15,53 | | | | | |
| | | **300 ms** | 3 | 11 | | _ | 1,04 | 0,03 | % | | treatment | treatment | | Control vs sAη-α | p>0,9999 | t(84)= 0,08895 | |
| | | | 3 | 12 | | sAη-α | 1,03 | 0,05 | % | | p=0,7824 | F (1, 63) = 0,0769[illegible] | | | | | |
| RjOrl:SWISS | **I/O** | **0.1 mV** | 4 | 7 | EPSP slope | _ | 0,117634262 | 0,0140231 | % | Two-way ANOVA | interaction p=0,9974 | F (3, 48) = 0,01519 | Sidak's | Control vs.Control | p=0,9896 | t(48)=0,4139 | **S1b** |
| | | | 4 | 7 | | _ | 0,09444753 | 0,0225927 | % | | | | | | | | |
| | | **0.2 mV** | 4 | 7 | | _ | 0,231957891 | 0,023384 | % | | **time p<0,0001** | F (3, 48) = 19,02 | | Control vs.Control | p=0,9945 | t(48)=0,3496 | |
| | | | 4 | 7 | | _ | 0,212371057 | 0,0309529 | % | | | | | | | | |
| | | **0.3 mV** | 4 | 7 | | _ | 0,311017213 | 0,0435328 | % | | | | | Control vs.Control | p=0,9999 | t(48)=0,1302 | |
| | | | 4 | 7 | | _ | 0,303724585 | 0,0443224 | % | | retesting p=0,5680 | F (1, 48) = 0,3306 | | | | | |
| | | **0.4 mV** | 4 | 7 | | _ | 0,399198355 | 0,057836 | % | | | | | Control vs.Control | p=0,9984 | t(48)=0,2562 | |
| | | | 4 | 7 | | _ | 0,384845293 | 0,0559999 | % | | | | | | | | |
| | **I/O 10nM sAη–α** | **0.1 mV** | 3 | 8 | EPSP slope | _ | 0,129088906 | 0,0221622 | % | Two-way ANOVA | interaction p=0,9991 | F (3, 56) = 0,007462 | Sidak's | Control vs. sAη-α | p>0,9999 | t(56)=0,05298 | **S1c** |
| | | | 3 | 8 | | sAη-α | 0,132580932 | 0,0222377 | % | | | | | | | | |
| | | **0.2 mV** | 3 | 8 | | _ | 0,259067485 | 0,0403819 | % | | **time p<0,0001** | F (3, 56) = 24,28 | | Control vs. sAη-α | p=0,9997 | t(56)=0,1717 | |
| | | | 3 | 8 | | sAη-α | 0,270384224 | 0,0329775 | % | | | | | | | | |
| | | **0.3 mV** | 3 | 8 | | _ | 0,386970615 | 0,0543952 | % | | | | | Control vs. sAη-α | p>0,9999 | t(56)=0,02858 | |
| | | | 3 | 8 | | sAη-α | 0,385086387 | 0,0433801 | % | | treatment p=0,8757 | F (1, 56) = 0,02471 | | | | | |
| | | **0.4 mV** | 3 | 8 | | _ | 0,505709119 | 0,0714783 | % | | | | | Control vs. sAη-α | p>0,9999 | t(56)=0,1183 | |
| | | | 3 | 8 | | sAη-α | 0,513507936 | 0,0610509 | % | | | | | | | | |

## Table S2 - Statistics of Figure 2

| Mouse model and genetic background | Experiment | # mice | # slice | Measurement | Treatment | Average | S.E.M. | Units | Statistical Test | p value | F/t/z/R ETC value | Comparison | post-hoc test | p value | q(DF) value | Figure |
|---|---|---|---|---|---|---|---|---|---|---|---|---|---|---|---|---|
| RjOrl:SWISS | **LTP - recAη-α and recAη–β** | 5 | 8 | last 15 minutes | _ | 137,70 | 7,50 | % | One-Way ANOVA | **p=0,0106** | F(2,18)=0,4396 | control vs recAη-α | **Dunnett's test** | **0,0104** | q(18)=3,155 | **2d** |
| | | 2 | 6 | | recAη-α | 99,04 | 6,58 | % | | | | control vs recAη-β | | **0,0311** | q(18)=2,639 | |
| | | 3 | 7 | | recAη-β | 106,70 | 10,71 | % | | | | | | | | |

## Table S3 - Statistics of Figure 3

| Mouse model and genetic background | Experiment | # mice | # slice | Measurement | Treatment | Average | S.E.M. | Units | Statistical Test | p value | F/t/z/R ETC value | Comparison | post-hoc test | p value | q(DF) value | Figure |
|---|---|---|---|---|---|---|---|---|---|---|---|---|---|---|---|---|
| **RjOrl: SWISS** | **LTP - 100 nM sAη-NT and sAη–β–CT** | 8 | 11 | last 15 minutes | - | 127,30 | 8,03 | % | One_Way ANOVA | **p=0,0288** | f(2,22)=1,093 | control vs sAη-β-CT | Dunnett's test | 0,9955 | q(22)=0,081 | **3c** |
| | | 3 | 6 | | sAη-β-CT | 139,20 | 5,03 | % | | | | control vs sAη-NT | | **0,0285** | q(22)=2,638 | |
| | | 3 | 8 | | sAη-NT | 100,00 | 7,42 | % | | | | | | | | |
| | **LTP - 10nM sAη-NT** | 4 | 8 | last 15 minutes | - | 138,10 | 2,79 | % | Unpaired t test | **p=0,0051** | t=3,363; df=13 | - | - | - | - | **3e** |
| | | 3 | 7 | | sAη-NT | 124,00 | 3,18 | % | | | | | | | | |

**Table S4 – Statistics of Figure 4**

## Figure 4A-C

Mann Whitney U-Test

| | Baseline | Wash-In | p-value |
|---|---|---|---|
| **Aeta-Beta-CT (Figure 4A)** | 12 [8.5, 17.5] | 14 [9.5, 17.5] | 0.2 |
| **Aeta-Alpha (Figure 4B)** | 18 [11.1, 21.5] | 5 [1.3, 11.3] | 1.1e-23 |
| **Aeta-Beta (Figure 4C)** | 13 [9.5, 18.5] | 1.8 [0.5, 4] | 2e-33 |

*Values as Median [25th percentile, 75th percentile]*

## Figure 4D

Descriptives

| | Change from Baseline |
|---|---|
| **Aeta-Beta-CT** | 0.5 [-3, 4.5] |
| **Aeta-Alpha** | -9.3 [-14, -3.7] |
| **Aeta-Beta** | -10.5 [-15, -6] |

*Values as Median [25th percentile, 75th percentile]*

Kruskal-Wallis ANOVA Table

| 'Source' | 'SS' | 'df' | 'MS' | 'Chi-sq' | 'Prob>Chi-sq' |
|---|---|---|---|---|---|
| 'Groups' | 4609047.1 | 2 | 2304523.6 | 198.8 | 6.7e-44 |
| 'Error' | 7584742.4 | 524 | 14474.69 | | |
| 'Total' | 12193789.5 | 526 | | | |

Pairwise results from multiple comparison test (with Tukey-Kramer correction)

| Group A | Group B | Lower (95%) Confidence Interval | Estimate | Upper (95%) Confidence Interval | p-value |
|---|---|---|---|---|---|
| Aeta-Beta-CT | Aeta-Alpha | 140.9 | 178.1 | 215.3 | 9.6e-10 |
| Aeta-Beta-CT | Aeta-Beta | 164.3 | 202.2 | 240.2 | 9.6e-10 |
| Aeta-Alpha | Aeta-Beta | -16.1 | 24.1 | 64.3 | 0.3 |

## Table S5 - Statistics of Figure 5

| Rat model | Experiment | # rats | Measurement | Treatment | Average | S.E.M. | Units | Statistical Test | p value | F/t/z/R ETC value | Figure |
|---|---|---|---|---|---|---|---|---|---|---|---|
| **Sprague-Dawley** | **In vivo LTP** | 9 | first 10 minutes | sAη-α | 159,20 | 8,00 | % | Student t-test | **p=0,013** | t=2,915 df=14 | **5b** |
| | | 7 | first 10 minutes | sAη-β-CT | 192,20 | 5,34 | % | | | | |
| **Sprague-Dawley** | **In vivo LTP** | 9 | last 10 minutes | sAη-α | 166,00 | 10,60 | % | Student t-test | **p=0,024** | t=2,530 df=14 | **5c** |
| | | 7 | last 10 minutes | sAη-β-CT | 212,00 | 15,50 | % | | | | |
